# Validation of the Gender Congruence and Life Satisfaction Scale (GCLS) among the Finnish population—implementing a tool to measure outcomes for transgender health services

**DOI:** 10.1080/26895269.2024.2407626

**Published:** 2024-09-26

**Authors:** Niina Puustinen, Marko Salmenkivi, Kaisa Kettula, Bethany A. Jones, Katinka Tuisku

**Affiliations:** a Department of Psychiatry, Helsinki University Hospital and University of Helsinki, Helsinki, Finland; b NTU Psychology, School of Social Sciences, Nottingham Trent University, England, UK

**Keywords:** GCLS, gender diverse, gender dysphoria, gender incongruence, non-binary identities, scale, transgender, treatment outcome

## Abstract

**Background:**

Validated tools that can measure changes in gender dysphoria and are inclusive of all gender identities are needed. To date, there are no such tools that have been validated using the Finnish population. The aim was to perform a cross-cultural validation of the Gender Congruence and Life Satisfaction Scale (GCLS).

**Method:**

Data from the prospective 2019–2020 Helsinki Gender Identity Cohort (*N* = 773) were used to validate the GCLS for the Finnish population. The cohort individuals were asked to complete the GCLS and other validated psychological questionnaires as part of their clinical assessment and this register-based data were used. Confirmatory factor analysis (CFA) and Principal component analysis (PCA) were performed on the data to assess the factor structure of the GCLS in the Finnish cohort. Convergent validity of the GCLS was also evaluated.

**Results:**

The CFA did not show adequate fit and therefore PCA was conducted. The PCA recommended seven subscales with good internal consistency. Four factors related to gender congruence (genitalia, chest, social gender role recognition, other secondary sex characteristics), and three related to secondary outcomes (psychological well-being, social avoidance, and life satisfaction). The final three subscales factorized in a slightly different way in the Finnish cohort, compared to the seminal English validation of the GCLS. The correlation analysis showed good convergent validity of the factors retrieved by the PCA.

**Conclusion:**

The results show that the GCLS scale is a valid tool to measure dysphoria related to genitalia, chest, secondary sex characteristics, and social gender role recognition for the Finnish population.

## Introduction

Although not considered a mental health condition in itself (Richards et al., 2015), the 11th revision of the International Classification of Diseases (ICD-11) included “gender incongruence” as a diagnosis. Trans and gender diverse (TGD) people in Finland wanting to access gender-affirming treatments, which aim to alleviate gender distress, require a diagnosis according to the ICD criteria (previously ICD-10, and soon to be ICD-11). Concomitant with other Western countries (Kaltiala et al., 2019; Zucker, 2017), the number of individuals seeking gender-affirming treatment has increased 20-fold in Helsinki University Hospital from 2007 to 2017, and the increasing trend continues (Tuisku, 2023). Another significant trend has been the increased demand for gender-affirming treatments among individuals presenting with gender diverse identities (i.e. people who identify with a gender that is not male or female) (Kettula et al., 2019).

Gender-affirming treatment often includes hormonal treatment and surgery, such as mastectomy, removal of gonads, vaginoplasty, and phalloplasty. These treatments may require lifelong health care follow-up and can affect fertility (Coleman et al., 2022). It is important to note however, that some people who experience gender incongruence will not choose to access gender-affirming treatments (Cooper et al., 2020). This may be associated with a lack of gender or body distress, or other personal, social, or financial reasons.

A literature review for the Finnish national evidence-based medicine guidelines (Pasternack et al., 2019) showed that while individuals who had undergone gender-affirming treatments were mostly satisfied with the treatment outcome, there is a paucity of research that has explored the benefits and disadvantages of individual treatments. Thus, considering the increased demand for gender-affirming treatment, and the newly emerged need for these treatments among non-binary individuals (Kettula et al., 2019), research on the effectiveness and safety of gender-affirming treatments across core primary and secondary outcomes is necessary. In addition to this, the Finnish Institute of Health and Welfare will soon introduce the ICD-11 (to replace the ICD-10). New in the ICD-11, diagnostic labels for gender incongruence will be inclusive of all gender identities, and therefore, outcome measures that are also inclusive of gender diversity are needed.

To undertake this research, measures that are validated with the Finnish population and are able to robustly assess treatment outcomes are needed. Previously, gender-affirming treatments were evaluated using the Utrecht Gender Dysphoria Scale (Steensma et al., 2013) and the Hamburg Body Drawing Scale (Appelt & Strauß, 1988). These measures are not, however, completely suitable for measuring outcomes of gender care in accordance with the new ICD-11 diagnosis of gender incongruence, primarily because they are not inclusive of gender diversity. Progress was made when (Jones et al., 2019) developed the Gender Congruence and Life Satisfaction Scale (GCLS), which is suitable to assess treatment outcomes for non-binary individuals. The GCLS was developed with and for TGD people and was found to be a reliable and valid measure to assess primary (e.g. gender incongruence with the chest and genitalia) and secondary (e.g. psychological well-being) outcomes of gender-affirming care (Jones et al., 2019). Similarly, (McGuire et al., 2020) developed an all-gender-inclusive version of the Utrecht Gender Dysphoria Scale (UGDS-GS).

Gender dysphoria can be characterized as a multidimensional experience of very subjective nature (Cooper et al., 2020). In a recent systematic review concerned with the subjective nature of gender dysphoria, Cooper et al. (2020) identified gender incongruence as a key facet of gender dysphoria, but they noted that there was a complex interplay with social expectations and reactions to gender. For example, participants in the reviewed studies discussed the distress they experienced from being misgendered, as it heightened feelings of gender incongruence.

Typically, measures like the Hamburg Body Drawing Scale (Appelt & Strauß, 1988) have assessed gender incongruence from a one-dimensional perspective, only taking into account experiences associated with gender and body distress. While gender and body distress are important aspects of gender incongruence, psychosocial experiences also need to be considered. The GCLS (Jones et al., 2019), however, adopts a multidimensional approach for assessing gender incongruence, in line with the findings of Cooper et al. (2020) by including seven different subscales: psychological functioning (10 items), genitalia (six items), social gender role recognition (four items), physical and emotional intimacy (four items), chest (four items), other secondary sex characteristics (three items), and life satisfaction (seven items).

As there is no gold standard for measuring gender dysphoria across the spectrum of binary and non-binary gender incongruence, the validation of GCLS must be conducted by measures of structural validity (i.e. by testing the hypotheses behind the development of each factor and comparing them to a previously validated instrument measuring this factor): for example, in the case of GCLS, comparing the psychological functioning factor to the Beck Depression Inventory (BDI) (Beck et al., 1987) or Overall Anxiety Severity and Impairment Scale (OASIS) (Norman et al., 2006).

## The present study

Validated tools, inclusive of gender diversity and sensitive to the subjective and multidimensional nature of gender incongruence, are urgently needed to effectively evaluate the effectiveness of gender-affirming treatments in Finnish transgender health services. To address this, the aim of the current study was to test the factor structure and to determine the convergent validity of the GCLS scale among the adult Finnish TGD population.

## Methods

### Study setting

In Finland, according to legislation, the diagnosis of gender incongruence and gender-affirming interventions are available at two university hospitals: Helsinki University Hospital and Tampere University Hospital. In recent years, referrals to both university hospital adult units have been approximately equal in number, with approximately 600–900 people accessing each specialist setting per year (personal communication, Assistant Chief Medical Officer Uusi-Mäkelä, Tampere University Hospital). The current study was carried out in Helsinki University Adult’s Outpatient Clinic for Assessment of Gender Identity.

During the period 2019–2020, the 10th revised version of the World Health Organisations’ (WHO) International Classification of Diseases 10th revision (ICD-10) was in use at Helsinki University Hospital. According to the ICD-10, the criteria for gender incongruence (binary or non-binary) is persistent gender incongruence for a minimum duration of 2 years. The ICD-10 code F64.0 was used for individuals with persistent binary gender identity and clinical gender dysphoria. A diagnosis of other gender identity disorder (F64.8) was used for persistent non-binary gender identity with clinical gender dysphoria. Finally, gender identity disorder, not specified (F64.9), was used for individuals whose assessment was unfinished, or at the end, the gender dysphoria was secondary, etiologically ambiguous, or not attributable to stable and mature identity, or less than 2 years in duration. There are no published epidemiological data on the incidence of new F64.0 and F64.8 diagnoses in Finland during these years.

In collaboration with Dr. Jones, the GCLS questionnaire was in 2018 translated in Finnish and Swedish (the official languages in Finland) and re-translated to English for quality control. Since January 2019, GCLS has been used to support assessments in Helsinki University Outpatient Clinic for Assessment of Gender Identity. The examined data for the current validation study were obtained from the Helsinki University Hospital 2019–2020 Gender Identity Cohort.

### The 2019–2020 Helsinki Gender Identity Cohort

A naturalistic cohort was formed consisting of all the individuals with no confirmed diagnosis of F64.0 or F64.8, nor any previous gender-affirming treatments, and who had an appointment at the Helsinki University Hospital Outpatient Clinic for Assessment of Gender Identity (adult unit) between January 1, 2019 and December 31, 2020 (*N* = 773). All individuals who had their first appointment or treatment planning meeting in 2019 or 2020 were included in the cohort, as well as individuals who had not previously accessed gender-affirming treatments and came for a new evaluation. The clinical evaluation process during the first appointment and treatment planning meeting included a clinical psychiatric examination, and to complement and support assessment for gender incongruence, service users were also asked to complete a battery of questionnaires, which included the translated GCLS.

### Materials

During the assessment process, questionnaires were given to participants at two time points: during their first appointment and again during their treatment planning meeting. The questionnaires were similar, and consisted of questions concerning gender identity, gender dysphoria, body dysphoria, demographic information, functional capacity, mental well-being, and substance use. The scales used are described below:

#### Gender Congruence and Life Satisfaction Scale (GCLS) (Jones et al., 2019)

The GCLS was developed in 2018 for the newly emerged need for a validated tool to measure transgender healthcare outcomes in gender diverse populations (Jones et al., 2019). The tool includes 38 items, and participants are asked to rate their responses on a five-point Likert scale (11 items are reverse-coded). A higher score on the GCLS indicates greater gender congruence and better gender-related well-being, whereas a lower score indicates the opposite. In the primary validation study (Jones et al., 2019), the 38 items formed seven subscales, each of which had good (*α* > 0.7) internal consistency and the internal consistency for the global score was excellent (*α* = 0.95).

#### Beck Depression Inventory (BDI), 21 item version (Beck et al., 1987)

The BDI-21 was originally developed to measure intensity of depressive symptoms (Beck et al., 1987), and it is commonly used as a screening tool for depression. A validation study conducted in Finland showed that the BDI is useful in detecting depressive symptoms in the general population (Aalto et al., 2012). The 21 items in the BDI receive scores between 0 and 3, which sum up to a total score between 0 and 63. A higher score indicates more severe symptoms of depression. The BDI total score was used in the correlation analysis to evaluate convergent validity in this study. The internal consistency of the BDI-21 scale in the present data was good (*α* = 0.89).

#### Overall Anxiety Severity and Impairment Scale (OASIS) (Norman et al., 2006)

The OASIS is a five-item scale of anxiety symptoms, which has shown excellent one-month test–retest reliability, convergent validity and divergent validity (Norman et al., 2006). Each of the five items in the OASIS are scored between 0 and 4 (total score from 0 to 20). A higher score indicates more severe anxiety symptoms. In this study, the OASIS total score was used to assess convergent validity. The Cronbach’s alpha coefficient for OASIS in the present data was 0.86, inferring good internal consistency.

#### Health-Related Quality of Life (HRQoL) instrument (15-D^®^) (Sintonen, 2001)

The 15-D instrument is a standardized, validated measure of health-related quality of life that includes 15 questions covering 15 dimensions of HRQoL: mobility, vision, hearing, breathing, sleeping, eating, speech, excretion, usual activities, mental function, discomfort and symptoms, depression, distress, vitality, and sexual activity (Sintonen, 2001). Each dimension is divided into five levels of severity. Of these 15 dimensions, vision and hearing were used in this study to examine the reliability of correlation analyses when testing the convergent validity of the GCLS. The Cronbach’s alpha coefficient for 15-D in the present data was adequate (*α* = 0.75).

#### Short Warwick–Edinburgh Mental Well-Being Scale (SWEMWBS) (Stewart-Brown et al., 2009)

The SWEMWBS is a seven-item scale of mental well-being, which is a shortened version of the WEMWBS 14-item scale (Tennant et al., 2007). The short version has been shown to distinguish mental well-being between subgroups when examined using criterion-validity methods (Ng Fat et al., 2017). Each item on the SWEMWBS scale is scored between 1 and 5, with total scores, therefore, between 7 and 35. A higher score indicates better mental well-being. The SWEMWBS total score was used in this study to assess convergent validity of the GCLS. The internal consistency of the SWEMWBS scale in the present data was good (*α* = 0.85).

#### Body and social dysphoria

Participants were asked to fill out a visual analogue scale (VAS) from 0 to 100 to define the level of gender-related body dysphoria they are experiencing and a similar VAS from 0 to 100 to define their level of gender-related social dysphoria (i.e. to define how much distress they experience from their social gender roles).

Demographic information, including education, employment, living situation, and relationship status, was also collected from service users. The language of the questionnaire was decided according to the individual’s self-reported strongest language (Finnish 93.8%, Swedish 3.6% or English 2.5%—English version of the questionnaire in Supplementary material).

#### Procedure

The filled questionnaires were scanned into the Helsinki University Hospital electric patient information system, as the primary purpose of the collected information was clinical decision-making and clinical follow-up. The collected questionnaires were notified to the research secretary (T.H.), who formed an anonymized research register of the included individuals for the purpose of this 2019–2020 cohort study. In total, 755 individuals were included in the cohort personal register. The completeness and accuracy of the cohort personal register was reviewed after 31^st^ December 2020 as follows:

- *Removal of individuals mistakenly included in the cohort personal register:*
  Baseline clinical data, including possible psychiatric comorbidity and data on the treatment plan, were recorded in an anonymized form from patient records of the individuals in the cohort register by a research assistant (N.A.). As the patient records were thoroughly reviewed, 27 individuals were removed from the 2019 to 2020 cohort, as it was found that they were already diagnosed with gender incongruence abroad by a medical professional or had already received gender-affirming treatments abroad. Also, individuals who had applied for treatment because they regretted previous gender-affirming treatments were removed from the cohort personal register (*n* = 20). Individuals who had not had any medical evaluation for gender incongruence or gender-affirming treatment but had made their own choice to directly order hormonal medication online were still included in the cohort personal register (as they were undergoing medical evaluation for the first time).
- *Completing the cohort personal register:*
  To review the completeness of the cohort personal register, N.P. went through a list of all individuals who had an appointment during the 2019–2020 period. Via this, another 65 individuals were found who fulfilled the original inclusion criteria, and they were added to the cohort personal register (questionnaire not filled for some reason, for example remote appointment due to the Covid-19 pandemic).

**Figure 1.**
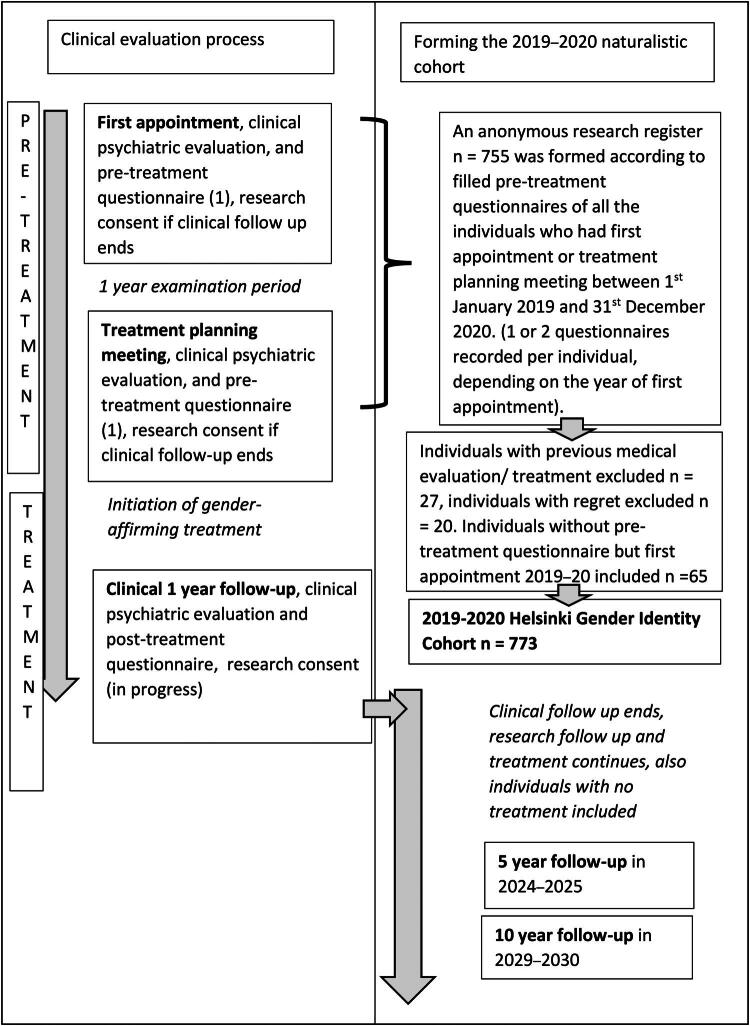
(GCLS). The X University Hospital 2019–2020 Gender Identity Cohort. This is a naturalistic cohort consisting of all individuals who did not have previous medically evaluated gender-affirming treatments and who had an appointment in X University Hospital Outpatient Clinic for Assessment of Gender Identity between January 1, 2019 and December 31, 2020. (1) contents of the questionnaire are shown in Supplement 1.

The compiling of the 2019–2020 Helsinki Gender Identity Cohort (*n* = 773) and the manner in which clinical data were collected is reviewed in Figure 1. The 2019–2020 Helsinki Gender Identity Cohort will be followed up for 10 years. The current validation study is based on baseline pretreatment data of the cohort, which should permit reliable follow-up of treatment outcomes in the future (Figure 1).

**Figure 2.**
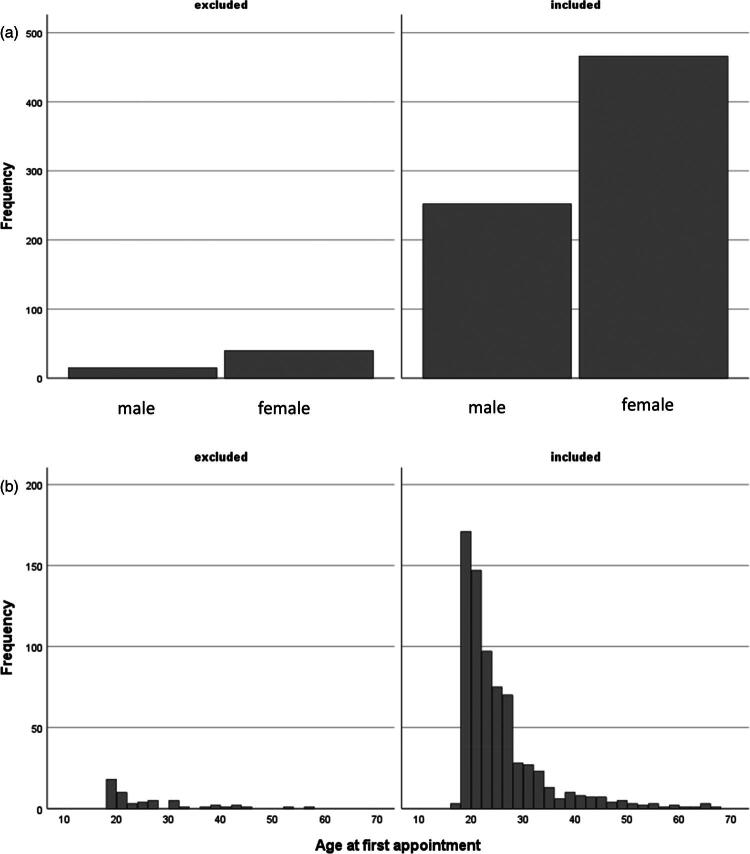
(a) Comparison of gender assigned at birth between excluded (no data available) and included (data available) study subjects. Mean difference: 0.77, 95% CI (−0.05, 0.20), independent samples *T*-test. (b) Comparison of age between excluded (no data available) and included (data available) study subjects. There is no significant difference between groups (*p* = 0.89 Mann–Whitney *U*-test).

In short, the GCLS and other questionnaire data were collected from the individuals included in the Helsinki 2019–2020 Gender Identity Cohort at two time points before they received gender-affirming medical treatment: at the first appointment and at the treatment planning meeting. At the treatment planning meeting it was concluded whether the criteria for ICD-10 diagnosis F64.0 (transsexualism) or F64.8 (other gender identity disorder) were met. For this validation study, GCLS data were available for 492/773 individuals from the first appointment, 476/773 individuals from the treatment planning meeting, and 718/773 individuals altogether before gender-affirming treatment (data either from the first appointment or the treatment planning meeting). Nine individuals were removed from the principal component analysis of GCLS, because there were four or more missing responses on the GCLS. When individuals with the pretreatment GCLS data available (*n* = 709) were compared to those who did not have this data available (*n* = 64), there was no statistical difference in age or gender assigned at birth (Figure 2(a,b)). In the following validation analyses, the data from the treatment planning meeting were primarily used; for those who did not have this data available, the data from the first appointment were used.

### Statistical methods

Confirmatory factor analysis (CFA) was run to determine the fit of the Helsinki Gender Identity Cohort data with the factor structure presented in the original validation study (Jones et al., 2019). The missing values (1.3%) were imputed to correspond to the median answers, where half numbers were rounded up. Because the Likert scale answers are ordinal, diagonally weighted least squares (DWLS) estimator was used instead of the maximum likelihood (ML) estimator (Xia & Yang, 2019).

As the CFA provided inadequate fit to the previously assumed factor structure, principal component analysis (PCA) was conducted to explore the factor structure of the GCLS in the Finnish sample. It was expected that the items of the GCLS would be related, and so therefore, oblique rotation (direct oblimin criterion) was employed. A scree test was conducted to determine the statistically significant factors to retain in the PCA.

To test the convergent validity of the factors retrieved by the PCA, Spearman’s Rho correlations with related scales (body dysphoria visual analogue scale, social dysphoria visual analogue scale, BDI, OASIS, SWEMWBS) were calculated. To assess the discriminant validity, we also calculated correlations with the 15-D items vision and hearing. Spearman’s Rho was chosen, as the data were not normally distributed. For the correlation analyses, the sum of the individual questions in each GCLS factor retrieved by the PCA was calculated to get the factor sum (items were reverse scored when needed). As multiple comparisons were conducted, Bonferroni corrections were used (0.05 ÷ 8 = 0.006). All statistical analyses were performed in R.

## Results

### Confirmatory factor analysis

The original seven factor model (Jones et al., 2019) demonstrated a moderate fit across fit indices: Comparative Fit Index (CFI) 0.956, Tucker-Lewis Index (TLI) 0.952, Root Mean Square Error of Approximation (RMSEA) 0.089 (95% CI 0.086–0.091), Standardized Root Mean Square Residual (SRMR) 0.089. According to Xia and Yang (2019), the use of the DWLS estimator causes overestimation of these indices, which leads to the conclusion that the CFI and TLI do not show adequate fit to the Finnish data. The RMSEA and SMSR below 0.08 would indicate a good fit to the data, which was not realized in this analysis. Therefore, it can be concluded that the proposed seven factor model did not fit very well to the Finnish data, and principal component analysis was conducted to examine the factor structure further.

### Principal component analysis

Using the scree plot of the explained variance is a common method to select the number of components for a PCA. However, when applied to our data, the scree plot did not indicate a clear number of components. Instead, increasing the number of components resulted in gradually decreasing increments of the explained variance. Eigenvalues greater than one were found for 14 principal components, and the corresponding model explained 76% of the total variance.

When choosing seven factors, similarly to the assumed factor structure of the GCLS (Jones et al., 2019), the model explained 60% of the total variance with 38 items (68% in Jones et al., 2019). The seven-factor model was chosen to display the results, as the internal consistency of the factors as measured by Cronbach’s alpha was better in this model than in the 14-factor model, and therefore, the internal consistency of this model was adequate. The seven-factor model is also simpler and it permits comparison to the original validation study (Jones et al., 2019). The seven-factor model is displayed in Table 1.

**Table 1.** Principal component analysis with seven factors.

Item	F1	F2	F3	F4	F5	F6	F7
Factor 1—Genitalia							
**21** I have been dissatisfied with my genitals as they do not correspond to my gender identity	**0.86**	0.00	−0.07	0.07	−0.01	0.16	−0.05
**26** I have felt that genital surgery would alleviate my dissatisfaction with my gender	**0.85**	0.06	−0.12	−0.11	0.02	0.08	−0.03
**29** I have been extremely distressed when looking at my genitals	**0.83**	−0.03	0.09	0.11	0.05	0.02	0.04
**14** I have been distressed when touching my genitals, as they do not match to my gender identity	**0.82**	−0.01	0.12	0.08	0.02	0.02	0.07
**27** I have not been able to live a satisfying life because of the distress related to my genitals	**0.79**	0.09	0.07	0.02	−0.01	−0.02	−0.02
**25** I have felt that my genitals correspond to my gender identity	**−0.63**	0.15	0.00	−0.13	−0.02	−0.12	0.11
Factor 2—Psychological well-being							
**9** I have felt that life is meaningless	0.10	**0.78**	0.01	−0.06	−0.06	−0.04	0.03
**13** I have thought about hurting myself or committing suicide	0.13	**0.71**	−0.02	−0.11	−0.04	−0.14	0.26
**10** I have not enjoyed life	0.03	**0.64**	0.19	0.12	−0.03	0.04	−0.14
**12** I have suffered from low mood	−0.07	**0.63**	0.17	0.20	0.01	0.15	0.00
**37** I have not been satisfied with my health	−0.12	**0.54**	−0.07	0.17	0.04	0.04	−0.02
**35** I have not been satisfied with my friends	0.05	**0.45**	−0.02	−0.05	−0.18	−0.04	0.05
**4** I have been anxious	−0.05	**0.43**	0.28	0.31	0.02	0.09	0.13
**31** I have been satisfied at school/lectures/work	0.14	**−0.39**	−0.22	−0.06	−0.05	−0.27	0.21
***8*** *I have thought about cutting or hurting my chest, genitals and/or surrounding areas*	0.28	**0.33**	0.07	−0.05	−0.15	*−0.37*	*0.42*
Factor 3—Social avoidance							
**1** I have avoided social situations and/or social interactions	−0.01	0.02	**0.76**	0.03	−0.05	0.07	0.06
**3** I have not been able to have close relationships with other people	0.16	−0.07	**0.74**	−0.06	−0.10	−0.09	−0.20
**7** I have had difficulty in making friends	−0.03	0.07	**0.69**	−0.03	−0.04	0.00	−0.11
**5** I have not been able to be physically intimate with other people	0.25	−0.04	**0.65**	−0.01	−0.08	−0.13	−0.09
**11** I have not participated in leisure activities	−0.05	0.16	**0.64**	0.04	−0.02	0.00	−0.09
**6** I have been unable to leave the house	−0.05	0.30	**0.57**	−0.06	0.06	0.12	0.24
**2** I have not gone to school/lectures/work	−0.08	0.23	**0.46**	−0.07	0.08	0.24	0.26
Factor 4—Chest							
**18** I have felt that my chest does not match to my gender identity	0.04	0.04	−0.12	**0.88**	−0.04	0.01	−0.06
**30** I have been satisfied with my chest	0.00	0.02	0.15	**−0.81**	0.09	0.05	0.12
**28** I have been extremely distressed when looking at my chest	0.18	0.05	0.13	**0.72**	0.01	−0.14	0.19
**15** I have been so distressed about my chest that I have not been able to live a fulfilling life	0.15	0.09	0.27	**0.62**	0.03	−0.16	0.16
Factor 5—Social gender role recognition							
**16** I have been satisfied with how others perceive my gender	0.05	0.01	−0.03	−0.06	**0.80**	−0.06	0.04
**20** I have been satisfied with the pronouns used by others to refer to me (in languages with gender pronouns, e.g. he/she in English)	−0.01	−0.05	0.12	0.09	**0.80**	0.10	−0.04
**22** I have been satisfied with how others perceive my gender based on my physical appearance	0.10	0.03	−0.07	−0.07	**0.74**	−0.16	0.07
**19** I have been distressed because others do not address me according to my gender identity	−0.01	0.02	0.24	0.29	**−0.48**	0.06	0.24
Variance explained (%)	**12**	**12**	**11**	**8**	**6**		
Eigenvalue	**4.58**	**4.47**	**4.29**	**3.08**	**2.46**		
Cronbach’s alpha	**0.91**	**0.80**	**0.84**	**0.83**	**0.74**		
Factor 6—Other secondary sex characteristics							
**24** I have felt that my facial hair does not correspond to my gender identity, either because I have it and I do not like it, or because I would like to have facial hair	0.24	0.02	−0.02	−0.11	−0.04	**0.78**	0.03
**17** I have felt that my body hair does not match to my gender identity, either because I have it and I do not like it, or because I would like to have body hair	0.21	0.06	−0.02	−0.15	−0.10	**0.77**	0.03
**23** I think that my voice has influenced the way others perceive my gender, and it has been distressing to me	0.02	−0.14	0.32	0.29	−0.17	**0.42**	0.16
Factor 7—Life satisfaction							
**32** I have been satisfied with my close relationships	−0.04	−0.27	−0.25	0.13	0.13	0.12	**0.56**
**33** I have been satisfied with my sex life	−0.38	−0.03	−0.18	0.08	0.07	0.04	**0.53**
***34*** *I have been satisfied with my leisure activities and hobbies*	0.04	*−0.47*	−0.16	−0.12	−0.05	−0.01	**0.42**
**36** I have been satisfied with the support I have received from close friends and family	−0.02	−0.18	−0.14	0.16	0.17	0.11	**0.40**
***38*** *I have been satisfied with my life in general*	0.02	*−0.71*	0.04	−0.05	0.10	−0.07	**0.33**
Variance explained (%)						**5**	**5**
Eigenvalue						**2.01**	**1.80**
Cronbach’s alpha						**0.73**	**0.70**

Factor loadings, eigenvalues, Cronbach’s alpha, and percentage of variance each factor explains (*n* = 709). *F*: factor.

*Italics* = items not placed according to their highest item loading.

Bold values = items retained in each factor.

The individual GCLS items were divided into the seven separate factors based on the highest item loading (positive or negative), and as a result, theoretically and clinically logical factors were found. Three exceptions were made: items 8, 34, and 38 were not placed according to their highest item loading; it was found that item 8 fitted better conceptually to factor 2 (psychological well-being) and items 34 and 38 to factor 7 (life satisfaction). These three exceptions also resulted in better internal consistency of the seven-factor structure, as measured by Cronbach’s alpha. All item loadings were above 0.30, as suggested adequate (Stevens, 2002). The factors related to gender congruence (genitalia, chest, social recognition, other secondary sex characteristics) included the same items as in the seminal study of Jones et al. (2019). These factors explained the overall variance as follows: “genitalia” (six items) explained 12%, “chest” (four items) explained 8%, “social gender role recognition” (four items), explained 6%, “other secondary sex characteristics” (three items) explained 5% (see Table 1).

The remaining 21 items factorized in a different way to Jones et al. (2019) study. Like Jones et al. (2019), we identified factors that conceptually assessed “psychological well-being” (nine items, 12%) and “life satisfaction” (five items, 5%), although the items included were slightly different (see Table 1). It was felt that the final factor conceptually captured “social avoidance” (seven items, 11%). This was not a factor identified in Jones et al. (2019) seminal development and validation study, and instead, their seventh factor assessed “physical and emotional intimacy.”

Together, factors 1 “genitalia,” 4 “chest,” and 6 “other secondary sex characteristics” describe the direct bodily dysphoria that the individuals seeking gender-affirming treatment experience and explain 25% of the overall variance. When social gender role recognition (6%) is added to this, it can be said that items related to gender dysphoria explain around half of the overall variance explained by the seven-factor model (31% vs. 60% overall). Factor 2 “psychological well-being,” which is directly related to mental health, explained 12% of the variance. Dissatisfaction with different aspects of social performance and life in general, which are conceptually more difficult to define, explained 16% of the overall variance, including factors 3 “social avoidance” and 7 “life satisfaction.” The exact items responded to by the study participants are displayed in Table 1.

### Construct validity

The convergent validity of the retrieved factors was tested using the additional data from the questionnaires that also included the GCLS scale and were therefore filled simultaneously. Results of the correlation tests are displayed in Table 2. After Bonferroni correction, *p* values < 0.006 were considered significant (eight correlations were tested).

**Table 2.** Spearman’s Rho correlations for the GCLS factor sums and related scales.

	correlation coefficient (CC)	95% CI	*p*
*F1 genitalia* vs. Body dysphoria VAS	0.46	0.40–0.52	< 0.001
*F2 psychological well-being* vs. BDI	0.58	0.52–0.64	< 0.001
*F2 psychological well-being* vs. OASIS	0.54	0.47– 0.61	< 0.001
*F3 social avoidance* vs. OASIS	0.47	0.41–0.53	< 0.001
*F4 chest* vs. Body dysphoria VAS	0.62	0.57–0.67	< 0.001
*F5 social gender role recognition* vs. Social Dysphoria VAS	−0.32	−0.39– −0.25	< 0.001
*F6 other secondary sex characteristics* vs. Body dysphoria VAS	0.22	0.14–0.29	< 0.001
*F7 life satisfaction* vs. SWEMWBS	0.58	0.53–0.63	< 0.001
*F1 genitalia vs. 15-D_Q2* *	0.02	−0.06–0.10	0.61
*F4 chest vs. 15-D_Q3* **	−0.04	−0.11–0.04	0.37

* 15-D Health-related quality of life question 2: vision.

** 15-D Health-related quality of life question 3: hearing.

Subscales 1 “genitalia,” 4 “chest,” and 6 “other secondary sex characteristics” were hypothesized to positively correlate with body dysphoria. When the correlation of the body dysphoria VAS was tested against the subscales “genitalia,” “chest,” and “other secondary sex characteristics,” the highest correlation was found with the “chest” subscale, with a correlation coefficient (CC) of 0.62. A notable correlation was also found with the “genitalia” subscale (CC 0.46). Correlation between the body dysphoria VAS and the “other secondary sex characteristics” subscale was low (CC 0.22), which may be because the VAS scale has only one item.

When the correlation between the social dysphoria VAS was tested against the sum of the “social gender role recognition” subscale, it was found that the correlation coefficient was low (CC −0.32). This negative CC was expected, as in the GCLS questionnaire, higher points mean higher satisfaction with gender role recognition, whereas in the social dysphoria VAS, higher points mean higher dysphoria.

The “psychological well-being” subscale correlated with BDI with a CC of 0.58, and with OASIS with a CC of 0.54. The “social avoidance” subscale was hypothesized to correlate with overall anxiety (OASIS), and the CC between these two was 0.47. Finally, the correlation of the “life satisfaction” subscale was tested against the Short Warwick Edinburgh Mental Well-Being Scale (SWEMWBS) and resulted in a CC of 0.58.

To assess discriminant validity, Spearman’s Rho correlations of items that should not correlate were tested (subscale “genitalia” vs. 15-D item vision, “chest” subscale vs. 15-D item hearing) (see Table 2); such correlations were very close to 0.

## Discussion

With the rising demand for gender-affirming treatment in the Nordic countries, there is an urgent need for a validated tool to measure changes in gender dysphoria before and after gender-affirming treatment to measure the health benefits of transgender healthcare. In the near future, the Finnish Institute of Health and Welfare will introduce ICD-11, which is inclusive of all gender identities. The change in diagnosis criteria used in the assessment of gender care, together with the current inclusive treatment practices (Coleman et al., 2022), requires outcome measures that are also inclusive of gender diversity, such as the GCLS. In light of this, the aim of the current study was to validate the GCLS with a Finnish adult transgender population sample.

The current study showed that the GCLS items (numbered 14–30) directly related to body dysphoria and social dysphoria factorized identically in Finnish adults seeking medical gender-affirming treatment and the primary UK sample of individuals aged 17 and older collected from a transgender service and community sample (Jones et al., 2019). These directly dysphoria-oriented subscales (factors genitalia, chest, social gender role recognition, and other secondary sex characteristics) also had statistically significant correlations with direct dysphoria measurements from VASs, indicating that these subscales also reliably measure body dysphoria and social dysphoria in the Finnish adult transgender population.

In the Finnish cohort, these directly dysphoria-related items (in factors genitalia, chest, social gender role recognition, and other secondary sex characteristics) explained 31% of the overall variance in the data, whereas in the UK study population these items explained 21% of the overall variance. This may be caused by differences in the study populations; the Finnish cohort included only transgender individuals who were seeking gender-affirming medical treatment, whereas the UK study population was also formed of community transgender individuals who were not all seeking gender-affirming treatment and also included cisgender individuals. Among the cisgender individuals, the variation in body dysphoria and social dysphoria can be assumed to be small (only little or no dysphoria), which would lower the variance explained by these items in the UK study population. The proportion of the explained variance in both study populations was high for the factor “genitalia,” which explained 12% of the variance in the Finnish cohort and 9% in the UK study population. There is a clinically logical explanation for the high variance explained by the factor “genitalia”; in the Finnish cohort, the majority of the study subjects were trans men (see Figure 2(b)), and in a clinical setting, it has been found that many trans men do not present with high genital dysphoria, whereas others can experience extremely high genital dysphoria (especially dysphoria concerning menstruation and the uterus, clinical note).

Although the items measuring body dysphoria and social dysphoria (subscales genitalia, chest, social gender role recognition, and other secondary sex characteristics) factorized identically in the Finnish and the UK cohort, there were notable differences in the factor structure of the items measuring psychological functioning and general life satisfaction. To make this difference visible, we named these factors differently (see Table 1). Whereas in the UK sample there were factors labeled as “psychological functioning” (10 items, 39% of the variance), physical and emotional intimacy (four items, 5% of the variance), and life satisfaction (seven items, 3% of the variance) (total variance explained 47%), the same items (1–13, 31–38) in the Finnish sample formed different factors: “psychological well-being” (nine items, 12% of the variance), “social avoidance” (seven items, 11% of the variance) and “life satisfaction” (five items, 5% of the variance) (total variance explained 28%). It seems that the cross-cultural repeatability of the principal component analysis of the GCLS scale is not as good in items 1–13 and 31–38 as it is for the items concerning body dysphoria and social dysphoria—items 14–30. There are several possible reasons for this. Firstly, there may be cultural differences in certain concepts that these items measure. The tendency to, for example, start avoiding social situations due to gender dysphoria may be different due to differences in social tolerance of diversity in the UK and in Finland. The time at which data were collected may also influence responses. Data for the UK study were collected earlier, in 2017, when gender diversity was likely less visible. Furthermore, translation of the GCLS scale to Finnish and Swedish may have changed the tone of some of the questions. For example, intimacy, in UK, refers to sexual intimacy, but direct translation to the Finnish word “läheisyys” means all kinds of closeness between two people. Some of the differences found may have also been caused by the differences in the study populations examined; the Finnish data comprised only clinically examined individuals and did not include any individuals who defined themselves as cisgender, whereas the UK data also included individuals who filled the GCLS scale online and cis individuals. In addition, there was a marked difference in the mean age of the study individuals. In the Finnish data, the mean age of the study individuals was 24, whereas in the UK data, the mean age was 36. Younger individuals may react differently to feelings of gender dysphoria compared to older individuals who have already settled into adult life.

However, the correlation tests confirmed the convergent validity of the GCLS scale among the Finnish adult transgender population. High correlations were found between factor 2 “psychological well-being” and the BDI/OASIS, factor 4 “chest” and the body dysphoria VAS, and factor 7 “life satisfaction” and SWEMWBS (Table 2). Moreover, fully moderate correlations between factor 1 “genitalia” and the body dysphoria VAS, and factor 3 “social avoidance” and the OASIS were also found. The correlation between factor 5 “social gender role recognition” and the social dysphoria VAS was statistically significant, but weaker than expected. There are several possible reasons for this weaker correlation. Firstly, item 20 in factor 5 asks whether the person is satisfied with the pronouns used by others. In the Finnish language there are no gendered pronouns, and this item may have lowered the resultant correlation. In addition, social dysphoria is a very abstract concept, and as the Finnish study population was very young and the prevalence of autism-related traits was quite high among this population (clinical note), some individuals may have found these kinds of abstract questions difficult to answer. The correlation between factor 6 “other secondary sex characteristics” and the body dysphoria VAS was also statistically significant but quite low (0.22). The two items in factor 6 concerning dysphoria related to body hair (or lack of body hair) may have been especially difficult to design so they applied to everyone (transwomen, transmen, and non-binary individuals).

The design of this validation study had several advantages. Data from the 2019 to 2020 Helsinki Gender Identity Cohort, a prospective cohort designed to measure the effectiveness of gender-affirming treatment among binary and non-binary transgender individuals, was used. All the individuals in the 2019–2020 Helsinki Gender Identity Cohort were examined by transgender health professionals at the Helsinki University Hospital Outpatient Clinic for Assessment of Gender Identity, and therefore, it can be ruled out that the study population had any individuals with only transient gender dysphoria or individuals who might have given untruthful answers in an online questionnaire. All the filled questionnaires were dealt with during clinical examination, and the answers were used to back up clinical evaluation of the study individuals. The prospective study design of the 2019–2020 Helsinki Gender Identity Cohort made it possible to design the content of questionnaire so that it was also suitable for research purposes (i.e. other previously validated scales were filled at the same time alongside the GCLS). The biggest limitation in using the data from the 2019–2020 Helsinki Gender Identity Cohort was that all individuals in the cohort are 18 years or older, so there were no minors in the cohort. Therefore, one must be careful when generalizing these results to the adolescent population seeking gender-affirming treatment. Bowman et al. (2022) recently produced an update of the Gender Preoccupation and Stability Questionnaire—revised version 2 (GPSQ-2), which was also validated among adolescents (7% of the participants in Bowman et al. study were minors). However, the GPSQ-2 questionnaire has a very different emphasis to the GCLS. While the GCLS focuses on body dysphoria, social dysphoria, and psychological functioning and well-being, the GPSQ-2 focuses on preoccupation on gender issues and stability of gender experience, which can be seen as more relevant issues in adolescent than in adult transgender healthcare. The GPSQ-2 correlated highly (0.75) with the GCLS psychological functioning factor (Bowman et al., 2022); however in our validation study, the cross-cultural stability of this particular GCLS factor could not be confirmed.

In our analyses, it was not possible to define known-groups validity, as all the cohort individuals have not reached the treatment planning phase by the time the diagnosis of non-binary, binary transgender, or other gender dysphoria is established. Due to the prospective nature of the study design, the effect of the possible gender-affirming treatments on the GCLS score was not possible to define at this stage. These issues can be seen as the major limitations of this study, and they need to be revisited in future reports. In addition, the 2019–2020 Helsinki Gender Identity Cohort does not include any cis individuals, and therefore, Finnish reference values for cis individuals could not be determined.

In Finnish transgender services, the GCLS scale can be used in a clinical setting to go through different aspects of gender dysphoria in a systematic manner to better comprehend the unique experience of each individual with gender dysphoria. When ICD-11 diagnostic criteria are implemented in Finland, the new diagnosis of gender incongruence (comprising both ICD-10 F64.0 and F64.8) will not change this, because the GCLS is also suitable for non-binary individuals. As the GCLS questionnaire is quite long, shortening it might be considered for clinical purposes; it may be better to include only items concerning body dysphoria and social dysphoria that factorized identically with the original UK population examined when the questionnaire was developed. However, if a shortened version of the GCLS questionnaire is used, one must combine it with other validated questionnaires that measure psychological well-being and quality of life.

The GCLS scale was chosen as the primary measure of gender dysphoria for this prospective 2019–2020 Helsinki Gender Identity Cohort because it was meticulously validated, and at the time of the study design (end of 2018), it was the only dysphoria scale that was also suitable for non-binary individuals. Subsequently, in 2020, a revised version of the Utrecht Gender Dysphoria Scale (UGDS-GS), also suitable for non-binary and genderqueer individuals, was introduced (McGuire et al., 2020). The UGDS-GS has been validated, but before a cross-cultural validation study in the Nordic countries is performed, we recommend using the GCLS in Finnish adult gender dysphoria units (research use and clinical use) and in Finnish gynecology, plastic surgery, and phoniatric units (research use), because of the better understanding of the characteristics of the GCLS scale provided by this study. In addition, in the validation study of the UGDS- GS, the research population was very different from that in the Finnish clinical setting (Figure 2(a,b)); for example, the UGDS-GS validation study had 323/1005 study subjects whose gender was assigned as male at birth but who identified themselves as non-binary/genderqueer. In the Helsinki University Adult’s Outpatient Clinic for Assessment of Gender Identity, this is a minority group, and most non-binary individuals are assigned as female at birth (clinical note). Therefore, the results of the UGDS-GS validation study may not be generalizable to Finnish transgender healthcare.

## Conclusion

The results of this cross-cultural validation study show that the GCLS scale is a valid tool to measure dysphoria related to genitalia, chest, secondary sex characteristics, and social gender role recognition for the Finnish population.

## Supplementary Material

Supplement 1_corrected.docx
