# Supplementary material for: Validation of the Gender Congruence and Life Satisfaction Scale (GCLS) among the Finnish population—implementing a tool to measure outcomes for transgender health services
Source: Int J Transgend Health. 2024 Sep 26;27(2):674–88. doi: 10.1080/26895269.2024.2407626 (PMC13015073; doi:10.1080/26895269.2024.2407626)
Supplement: Supplement 1_corrected.docx [file WIJT_A_2407626_SM8908.docx]

**Questionnaire for patients arriving to management plan meeting**

Please fill in this questionnaire and bring it to your management plan meeting. At the meeting, we can discuss any unclear items or you can specify your answers if you wish.

**Name_____________________________________ social security number: ___________________**

**BACKGROUND INFORMATION**

1. **Age** ___
2. **What is the highest education or degree you have completed (or equivalent Finnish education)**

| 0 | No education |
| --- | --- |
| 1 | Comprehensive school |
| 2 | Vocational school/apprenticeship |
| 3 | Upper secondary school (high school) |
| 4 | Degree from a university of applied sciences |
| 5 | University degree |

1. **Which of the following options best describes your current professional situation?**

|  | Are you: |
| --- | --- |
| 1 | Employed or an entrepreneur |
| 2 | A student |
| 3 | Unemployed |
| 4 | On disability pension or rehabilitation allowance |
| 5 | On family leave or caring for a close relative |
| 6 | Other? |

1. **Who do you currently live with?**

| 1 | Alone |
| --- | --- |
| 2 | With a roommate/mates but not with a partner |
| 3 | With a partner |
| 4 | With a partner and a child/children |
| 5 | With a child/children without a partner |
| 6 | With my parents (and/or siblings) |
| 7 | Someone else, who? |

1. **Are you currently in a steady relationship?**

|  |  |
| --- | --- |
| Yes | No |

1. **Gender identity**

Please place an X on a point on the line that best corresponds to your gender identity.

Not male ______________________________________________ Male

0—10—20—30—40—50—60—70—80—90—100

Not female ______________________________________________ Female

0—10—20—30—40—50—60—70—80—90—100

Not other ______________________________________________ Other, please specify:  0—10—20—30—40—50—60—70—80—90—100

If you would have to describe your gender in one word, which of the following would you choose?

| 1 | Female | 6 | Agender | 11. Other, please specify? |
| --- | --- | --- | --- | --- |
| 2 | Male | 7 | Other-gendered |  |
| 3 | Trans-woman | 8 | Non-binary |  |
| 4 | Trans-man | 9 | Gender queer |  |
| 5 | Female and male | 10 | Gender fluid |  |

1. **Gender dysphoria**

Gender dysphoria means distress that is caused by a discrepancy between a person’s gender identity and that person’s sex assigned at birth (and the associated gender role and/or primary and secondary sex characteristics) Source: WPATH 2011.

How much distress do you experience from social gender roles?

Not at all 0—10—20—30—40—50—60—70—80—90—100 Intolerable

Please briefly describe the 1 to 3 most distressing situations in your everyday life

____________________________________________________________________________

____________________________________________________________________________

How much distress do you experience from body-related dysphoria?

Not at all 0—10—20—30—40—50—60—70—80—90—100 Intolerable

Which characteristic(s) of your body cause(s) you most distress?

____________________________________________________________________________

____________________________________________________________________________

____________________________________________________________________________

**Gender Congruence and Life Satisfaction Scale (GCLS)**

(Jones et al. 2019, see the reference in the main text, full questionnaire removed from this supplement).

**FUNCTIONAL CAPACITY**

How do you evaluate your functional capacity during the past month in the following areas of life

Professional functional capacity (at work, job seeking, studying)

not able to function 0—10—20—30—40—50—60—70—80—90—100 excellent

Social functional capacity (relationships, hobbies, running errands, participation)

not able to function 0—10—20—30—40—50—60—70—80—90—100 excellent

Personal functional capacity (taking care of personal basic needs and housekeeping)

not able to function 0—10—20—30—40—50—60—70—80—90—100 excellent

**QUALITY OF LIFE**

On a scale of 0 to 100, how would you evaluate your quality of life?

very poor 0—10—20—30—40—50—60—70—80—90—100 very good

**Health-related Quality of life questionnaire 15D©**

(Sintonen 2001, see the reference in the main text, full questionnaire removed from this supplement).

**HEALTH AND WELLBEING**

**Overall Anxiety Severity and Impairment Scale (OASIS)**

(Norman et al. 2006, see the reference in the main text, full questionnaire removed from this supplement).

**Beck Depression Inventory (BDI-21)**

(Beck, 1987, see the reference in the main text, full questionnaire removed from this supplement).

**ALCOHOL**

How many standard units of alcohol do you consume on average in a week?_______

How often do you consume more than 6 units* of alcohol?

| 0 | Never |
| --- | --- |
| 1 | Less than monthly |
| 2 | Monthly |
| 3 | Weekly |
| 4 | Every day or nearly every day |

| *) 1 unit = | 12 cl of wine | 1.25 units = | bottle of over 4.7% beer |
| --- | --- | --- | --- |
|  | 8 cl of fortified wine |  | bottle of over 4.7% cider |
|  | 4 cl of spirits or other strong alcohol |  | bottle of long drink |

?

Have you used intoxicating substances or drugs for non-medicinal purposes in the previous 12 months?

| Yes | No |
| --- | --- |

**Shor Warwick-Edinburgh Mental Well-Being Scale**

(Stewart-Brown et al, 2009, see the reference in the main text, full questionnaire removed from this supplement).

**THANK YOU FOR YOUR ANSWERS**

**Would you like to add something or do you have any comments?**

_________________________________________________________________________________

_________________________________________________________________________________

__________________________________________________________________________________
